# Anergic B Cells: Precarious On-Call Warriors at the Nexus of Autoimmunity and False-Flagged Pathogens

**DOI:** 10.3389/fimmu.2015.00580

**Published:** 2015-11-26

**Authors:** Allen J. Rosenspire, Kang Chen

**Affiliations:** ^1^Department of Immunology and Microbiology, Wayne State University, Detroit, MI, USA; ^2^Department of Obstetrics and Gynecology, Wayne State University, Detroit, MI, USA; ^3^Department of Oncology, Wayne State University, Detroit, MI, USA; ^4^Perinatology Research Branch, Eunice Kennedy Shriver National Institute of Child Health and Human Development, National Institutes of Health, Detroit, MI, USA; ^5^Tumor Biology and Microenvironment Program, Barbara Ann Karmanos Cancer Institute, Detroit, MI, USA; ^6^Mucosal Immunology Studies Team, National Institute of Allergy and Infectious Diseases, National Institutes of Health, Bethesda, MD, USA

**Keywords:** B cell, anergy, autoimmunity, infection, vaccines, immunotherapy of cancer

A tenet of modern immunology is that the adaptive immune system has evolved so as to prevent, or at least diminish responses targeting self-antigens (1). Self-reactive B cells that arise due to incomplete negative selection in the bone marrow have been shown to be removed or inactivated in the periphery, with the most strongly self-reactive cells subject to clonal deletion. Less self-reactive cells either undergo receptor editing or are rendered anergic (2, 3). Receptor editing entails the reactivation of recombinase activating genes (RAGs) and enables immunoglobulin genes to be rearranged to create new antigen specificities. Anergy is a poorly understood state whereby cells retain the ability to bind to self-antigens but are otherwise rendered insensitive to antigenic stimulation (4). However, anergy is not a perfect solution to control self-reactivity. Many multifactorial autoimmune disorders involve the disruption of B cell anergy as a potential mechanism (5). One of the best known examples is systemic lupus erythematosus (SLE), where the inappropriate activation of anergic B cells and their differentiation into plasma cells that secrete autoreactive antibodies are an important contributing pathogenic mechanism. In particular, a large number of the autoantibodies in SLE is of the 9G4 idiotype (6). It has been shown that 9G4 idiotypic B cells are present and anergic in normal individuals, but actively expand into the plasma and memory cell compartments in SLE patients (7).

Much effort has been devoted to delineating the molecular differences in the signaling pathways between anergic and normal B cells, in order to understand their difference in the ability to respond to antigenic stimulation. Multiple mechanisms are likely involved. For instance, anergic B cells differ from normal B cells in the functioning of several signaling elements down-stream of the B cell receptor, such as the tyrosine kinase Lyn and the tyrosine phosphatase SHIP-1 (4). It has also been shown that a population of human peripheral blood naive B cells expressing autoreactive IgD receptor but no IgM receptor is anergic (8), and the responsiveness of B cells to low valence antigens was decreased by the flexible hinge region of IgD (9), suggesting that switching the B cell receptor usage from IgM to IgD resulting in higher IgD/IgM ratios may be a means to achieve anergy.

Related studies have focused on the mechanisms that permit anergic B cell activation in the context of autoimmune diseases (7). Lost in the mechanistic discussions, however, has been the question as to why anergic B cells exist in the first place. After all, these are dangerous cells, which could have been easily removed by clonal deletion early on during B cell development. Their persistence in the periphery implies to us that they must serve some immunological function. Recently, studies on B cell exhaustion associated with certain chronic infection (10, 11) suggest that rescue from anergy by proper stimuli may in some cases be a necessary measure to fight infections. We agree that rescue from anergy may in some instances be necessary to fight infection, and further contend that the molecular underpinning of this phenomenon may involve the cross-reactivity of the pathogens with the host (Figure 1).

**Figure 1:**
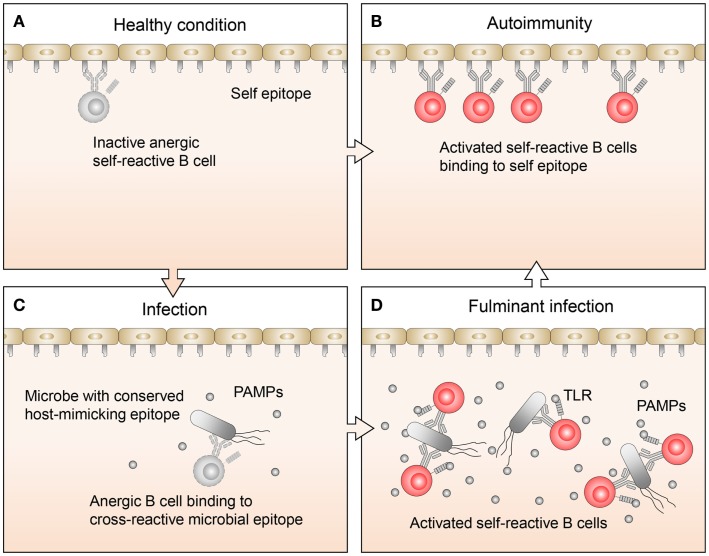
**Proposed regulation and function of anergic B cells**. **(A)** Under healthy conditions, there are a limited number of self-reactive B cells. These cells are mostly restricted to the anergic B cell pool, so while they may bind self-antigens, they are not activated. **(B)** In autoimmune disease, anergic B cells often bind to self-antigen, become activated, and respond by transitioning out of the anergic B cell pool, leading to an expansion of self-reactive B cells. **(C)** Normally during infections, B cells, which recognize pathogen-specific antigens clonally expand. If the pathogen also expresses host-mimicking epitopes, anergic B cells which recognize these epitopes may bind but are not activated because the levels of pathogen-associated molecular pattern (PAMP) molecules are low. **(D)** Under conditions where an infection becomes fulminant and PAMP levels are elevated, TLR signaling may synergize with signals from the BCR on anergic B cells, allowing them to transition out of the anergic B cell pool. This will lead to an expansion of activated B cells, which recognize host-mimicking epitopes on the pathogen, as well as antigens on host tissues.

Pathogens often have cross-reactive antigens with their host. It is for this reason that streptococci infections responsible for rheumatic fever can result in the production of autoantibodies targeting heart valves (12). Likewise, coxsackie virus infections can provoke the production of autoantibodies targeting the heart (13, 14). There are numerous other examples of the causal association between pathogen infection and autoantibody production. In fact, it is generally believed that viruses and bacteria are among the environmental triggers of autoimmune diseases (15). However, it has commonly been thought that when autoimmunity occurs as a result of infection, the appearance of cross-reactivity between pathogenic and self-antigens is serendipitous.

We propose that the appearance of antigenic mimicry between pathogens and their host is the result of an evolutionary adaptation, whereby pathogens protect themselves from the host’s immune response by co-evolving with their host vital antigenic epitopes with immunological similarity to host antigens. In other words, these vital pathogen epitopes are protected because they would not trigger the host’s immune system, as they appear to be self to the host. In a military analogy, they are wearing deceptive uniforms and are “false flagged.” It is our view that anergy reflects a counter-balancing host adaption enabling adaptive immunity to deal with “falsely flagged” pathogens. We suggest that anergic B cells are held in abeyance until such time as when they are absolutely needed to neutralize an infection and the issue of autoimmunity has become of secondary importance. In this way, anergic B cells would serve as a cellular reservoir that can be deployed when needed to expand the immune repertoire to include antibodies targeting vital epitopes on pathogenic organisms which are normally hidden from immune surveillance by cloaking themselves via cross-reactivity with antigens of the host. This view is in accord with a previous suggestion that anergic B cells, aside as serving as a reservoir of cells responsible for autoantibodies that characterize rheumatic diseases, may also serve a useful function for protective immunity (16).

This circle of ideas is perhaps best supported by recent experience with human immunodeficiency virus (HIV) where a connection between the ability to generate broadly neutralizing antibodies (BNAs) to HIV and autoimmunity has been found (17, 18). Almost from the beginning of the HIV epidemic, it has been observed that the incidence of HIV in SLE is significantly lower than that in the general population as a whole (19). Recently, it has been shown that this epidemiological finding can likely be explained by the fact that SLE seems to be linked to the ability to make BNAs to HIV (20). Interestingly, 9G4 autoreactivity, the marker for SLE as discussed above, is increased in HIV-infected patients, and correlates with HIV BNA activity, suggesting that production of BNAs is connected to a loss of tolerance (21, 22). Furthermore, it has also been found that naturally arising BNAs, whether from SLE patients or not, almost invariably possess some level of autoreactivity (23, 24). In fact, a recent review surveying over 120 BNAs reported that all of them were associated with relaxation of host tolerance (23). In a related vein, it has been found that genetic variants associated with psoriasis are also protective against HIV-1 disease (25). Although in this case the presence of BNA was not investigated, again the implication is that not just SLE, but autoimmunity in general may be associated with the ability to produce BNAs to HIV.

Recent findings show that BNAs, when they are produced tend to bind to only a few select epitopes of the HIV trimer (26). In the context of the autoimmune phenomena discussed above, the conclusion that we draw is that those determinants on HIV to which BNA are reactive are strategically important for virus function. We suspect that in general they are difficult for the immune response to target, precisely because the virus has evolved in such a way as to protect them by making these determinants cross-reactive with a host antigen. The idea that a pathogen can escape immune detection by mimicking host epitopes has recently been suggested to apply to bacterial pathogens with respect to capsular polysaccharides which are structurally similar to host polysaccharides (27). Accordingly, a prerequisite for initiating an immune response targeting cross-reactive epitopes is that the immune repertoire first be expanded to insure such epitopes are able to appropriately interact with a receptive B cell. We propose that the source of this expanded repertoire is the anergic B cell pool.

Following this argument, one might ask, how can anergic cells be part of the normal adaptive immune response if they are ultimately responsible for autoimmunity? However, as we have pointed out above, exposure to pathogens often does give rise to autoimmunity and autoimmune diseases! A more relevant question might be, if anergic cells are part of the normal adaptive immune response, why do not we see more autoimmune diseases? The answer would appear to be that this is precisely why anergic cells are held under such tight control. We assume that they would be only called upon as a last resort of contingency, when other cellular components of the immune system have failed to control an infection. At this point, the immediate concern of the host would likely be control of acute infection, with potential chronic autoimmune considerations of secondary importance.

The question arises as to how the immune system might call these reserve anergic cells into action when the need arises. Although this is an open question, we suggest that toll-like receptors (TLRs) will turn out to be an important part of the answer. It is well known that TLRs provide complementary stimulatory signals to B cells upon binding pathogen-associated molecules such as CpG DNA and LPS (28). It has been shown by multiple investigators that anergic cells respond to TLR4 stimulation, as anergy can be partially broken in human and mouse anergic B cells by exposure to LPS, albeit at higher concentrations than those needed to activate non-anergic B cells (29–34). More recently, it has been shown that signaling through TLRs other than TLR4 likely can also break anergy, as detection of nucleic acids by TLRs is also linked to SLE (35).

In any event, it is not too hard to envisage that when normal immune mechanisms fail to resolve infections, such as in a fulminant infection, high levels of TLR agonists, pathogen-associated molecular pattern (PAMP) molecules will result. We expect that it is only under conditions of abnormally high levels of PAMPs that anergic cells respond to TLR signaling, potentially overcoming quiescence in antigen-stimulated anergic B cells. In this way, it may be molecularly and immunologically possible to release anergy in a controlled fashion, only when needed. However, if this is true, then it would also be predicted that TLR stimulation of anergic B cells would also be associated with autoimmunity. In support of this idea, we note that TLRs and the MyD88 signaling pathway have been shown to play an essential role in the generation of autoantibodies in mouse models of autoimmunity (36–40). It has, in fact, been previously suggested that bacteria-derived LPS could circumvent normal tolerogenic controls and thereby contribute to the generation of autoimmune disease (34).

The association of TLRs with models of autoimmunity may represent situations where TLRs have been coaxed to aberrantly activate anergic B cells. Generally, we would expect that the activation of anergic B cells would not always lead to excessive autoimmunity. Although pathogenic antigens might be cross-reactive with host antigens, they would not be expected to be identical. In this case, affinity maturation in the germinal centers over time would be expected to drive the immune response to more closely target the pathogenic epitopes, rather than self. This idea is supported by findings that show that DNA sequences associated with BNA HIV antibodies are highly mutated from germ line sequences, as they are the products of multiple rounds of affinity maturation lasting of the order of perhaps 2 or more years (41–43).

Finally, experiments demonstrating association of TLRs with models of autoimmunity may provide insight into how we might activate anergic cells for useful therapeutic purposes. For instance, returning to the problem of developing an HIV vaccine, we might want to explore the use of targeting TLRs on anergic B cells as a way of expanding the immune repertoire prior to inoculations with HIV-specific antigens. Likewise in immunotherapy, tumor-associated antigens are in most instances not uniquely expressed on tumor cells, so that raising an immune response to a tumor is in many respects akin to raising an autoimmune response, although to a much more limited extent than is usually associated with autoimmunity. Nevertheless, if reactivity to tumor-associated antigens is represented in the immune repertoire of anergic cells, then activation of these cells through manipulation of TLRs prior to immunization with “tumor-specific” antigens might prove beneficial.

## Conflict of Interest Statement

The authors declare that the research was conducted in the absence of any commercial or financial relationships that could be construed as a potential conflict of interest.
